# Late-Life Depressive Symptomatology, Motoric Cognitive Risk Syndrome, and Incident Dementia: The “NuAge” Study Results

**DOI:** 10.3389/fnagi.2021.740181

**Published:** 2021-09-30

**Authors:** Olivier Beauchet, Harmehr Sekhon, Cyrille P. Launay, Pierrette Gaudreau, José A. Morais, Gilles Allali

**Affiliations:** ^1^Department of Medicine, University of Montreal, Montreal, QC, Canada; ^2^Research Center of the Geriatric University Institute of Montreal, Montreal, QC, Canada; ^3^Department of Medicine, Division of Geriatric Medicine, Sir Mortimer B. Davis Jewish General Hospital and Lady Davis Institute for Medical Research, McGill University, Montreal, QC, Canada; ^4^Lee Kong Chian School of Medicine, Nanyang Technological University, Singapore, Singapore; ^5^Research Center of the Centre Hospitalier de l'Université de Montreal, Montreal, QC, Canada; ^6^Division of Geriatric Medicine, McGill University Health Centre, Montreal, QC, Canada; ^7^Department of Neurology, Geneva University Hospital, University of Geneva, Geneva, Switzerland

**Keywords:** aging, community dwellers, dementia, depression, motor dysfunction, cohort study, EPI–epidemiology

## Abstract

**Background:** Late-life depressive symptomatology and motoric cognitive risk syndrome (MCR) have independently been associated with an increased risk for incident dementia. This study aimed to examine the association of late-life depressive symptomatology, MCR, and their combination on incident dementia in community-dwelling older adults living in Quebec (Canada).

**Methods:** The study was carried out in a subset of 1,098 community dwellers aged ≥65 years recruited in the “*Nutrition as a determinant of successful aging: The Quebec longitudinal study*” (NuAge), an observational prospective cohort study with 3 years follow-up. At baseline, MCR was defined by the association of subjective cognitive complaint with slow walking speed, and late-life depressive symptomatology with a 30-item Geriatric Depression Scale (GDS) score >5/30. Incident dementia, defined as a Modified Mini-Mental State score ≤79/100 test and Instrumental Activity Daily Living score <4/4, was assessed at each annual visit.

**Results:** The prevalence of late-life depressive symptomatology only was 31.1%, of MCR only 1.8%, and the combination of late-life depressive symptomatology and MCR 2.4%. The combination of late-life depressive symptomatology and MCR at baseline was associated with significant overall incident dementia (odds ratio (OR) = 2.31 with *P* ≤ 0.001) but not for MCR only (*OR* = 3.75 with *P* = 0.186) or late-life depressive symptomatology only (*OR* = 1.29 with *P* = 0.276).

**Conclusions:** The combination of late-life depressive symptomatology and MCR is associated with incident dementia in older community dwellers. The results suggested an interplay between late-life depressive symptomatology and MCR exposing them to an increased risk for dementia.

## Background

Late-life depressive symptomatology is a common psychiatric condition in older adults with a prevalence ranging from 8 to 16% (Diniz et al., 2013; Bennett and Thomas, 2014; Steffens, 2017). A systematic review and meta-analysis of community-based cohort studies have shown that late-life depressive symptomatology is associated with an increased risk for incident dementia, regardless of its type (i.e., Alzheimer's disease (AD) and non-AD) (Diniz et al., 2013). Older adults with late-life depressive symptomatology frequently have subjective cognitive complaints (SCC) and a slow walking speed compared to their healthy counterparts (Lichtenberg et al., 1995; Bennett and Thomas, 2014). The combination of these two clinical characteristics (i.e., SCC and slow walking speed) defines the motoric cognitive risk syndrome (MCR), which is associated with an increased risk for dementia (Verghese et al., 2013). Recently, it has been reported in the Canadian population that there was a higher prevalence of depressive symptomatology in adults with MCR compared to those without MCR (Sekhon et al., 2019a). We suggested that this overlapping condition influences the risk for incident dementia. More precisely, we hypothesized that the presence of late-life depressive symptomatology with MCR could increase the risk for incident dementia, regardless of its type, compared to each condition alone. We used the data collected in the “*Nutrition as a determinant of successful aging: The Quebec longitudinal study*” (NuAge) to test this hypothesis (Gaudreau et al., 2007). NuAge is a 3-year-follow-up observational prospective cohort study performed in community dwellers aged ≥65 years living in Quebec (Canada). This study aimed to investigate the association of late-life depressive symptomatology, MCR, and their combination with incident dementia in participants of the NuAge study.

## Methods

### Population

The subset of NuAge participants selected for this study were participants without mobility disability defined by using a walking aid; with information on walking speed and depressive symptomatology assessed with the 30-item Geriatric Depression Scale (GDS) at the baseline assessment; and with information on their cognitive status performance using the Modified Mini-Mental State (3MS) and the simplified instrumental activity daily living (IADL) scores using items of the Functional Autonomy Measurement System (SMAF) over the 3-year follow-up (Yesavage et al., 1982–1983; Teng and Chui, 1987; Hebert et al., 1988; Pérès et al., 2006; Gaudreau et al., 2007). Participants lost to follow-up and those who withdrew their agreement to use data were also excluded. A total of 1,098 (62.6%) NuAge participants from the full set of 1,754 participants were selected.

### Assessment

Age, sex, the number of medications, height (m), weight (kg), history of falls, walking speed (m/s), simplified IADL score, 3MS score, and 30-item GDS score were recorded at the baseline assessment (Yesavage et al., 1982–1983; Teng and Chui, 1987; Hebert et al., 1988; Pérès et al., 2006). The Physical Activity Scale for the Elderly (PASE) was used to determine the level of physical activity at baseline; a low level was defined by being below the lowest tertile (i.e., <69.1 for female and <87.7 for male) (Washburn et al., 1999). The body mass index (BMI; kg/m^2^) was also determined at baseline; overweight and/or obese were considered if BMI was ≥25 kg/m^2^. Polypharmacy was defined as ≥5 drugs taken daily. The 3MS and simplified IADL scores were recorded annually over the 3 years of follow-up (Yesavage et al., 1982–1983; Hebert et al., 1988; Pérès et al., 2006).

### Definition of Late-Life Depressive Symptomatology, MCR, and Dementia

The late-life depressive symptomatology was defined at baseline as a 30-item GDS score >5/30 (Yesavage et al., 1982–1983; Alexopoulos et al., 2005). MCR was defined by the combination of SCC with slow walking speed in the absence of major neurocognitive disorders and motor disability (Verghese et al., 2013). The answer “Yes” to the item “*Do you feel you have more problems with memory that most*?” of 30-item GDS defined SCC. A walking speed of one standard deviation (SD) or more below the age-appropriate mean values in the selected subset of participants defined slow walking speed. Cut-off values for defining slow walking speed were calculated as described by Verghese et al. (2013). Overall, the incident dementia was defined with a 3MS score ≤ 79/100 and simplified IADL score <4/4 at each annual visit after the baseline assessment over the 3-year follow-up (Teng and Chui, 1987; Hebert et al., 1988; Pérès et al., 2006). Low education was associated with a greater risk for incident dementia (Sharp and Gatz, 2011). The number of years of education has been collected in the NuAge cohort and used as a measure of education. There was no significant difference in the number of years of education between groups (data not shown).

### Standard Protocol Approval and Participant Consents

The NuAge protocol was approved by the Research Ethics Board (REB) of the University Institutes of Geriatrics of Sherbrooke and Montreal (Quebec, Canada), and the NuAge Database was approved by the REB of the CIUSSS-de-l'Estrie-CHUS (Quebec, Canada). All the participants signed consent for research. The REB of the Jewish General Hospital (Montreal, Quebec, Canada) approved the present study.

### Statistical Analysis

Participants were separated into four groups using the baseline assessment information: (1) no late-life depressive symptomatology and no MCR, this group was used as the reference group; (2) late-life depressive symptomatology only (i.e., being in the group of participants with late-life depressive symptomatology only means that participants with late-life depression and MCR are excluded from this group); (3) MCR only (i.e., being in the group of participants MCR only means that participants with MCR and late-life depression and MCR are excluded from this group); and (4) combination of late-life depressive symptomatology and MCR. The characteristics of the participants were described using means, SD, and percentages. The ANOVA, Kruskal–Wallis, unpaired *t*-test, Mann–Whitney, Chi-square test, or Fisher's exact tests were used for group comparisons, as appropriate. Value of *P* < 0.01 was considered statistically significant because of multiple comparisons (Verghese et al., 2013). Multiple logistic regressions examined the association of overall incident dementia (dependent variable) with late-life depressive symptomatology, MCR, and their combination (independent variables in separated models) with adjustment for baseline characteristics of the participants. Cox regression models were not used because only the annual incidence of dementia was collected and not the exact date of diagnosis of dementia. Furthermore, the follow-up period was short and limited to 3 years. Value of *P* < 0.05 were considered statistically significant for logistic regressions. Statistics were performed using SPSS (version 24.0).

## Results

At baseline, the prevalence of having only late-life depressive symptomatology, MCR, or the combination of both late-life depressive symptomatology and MCR was 31.1, 1.8, and 2.4%, respectively. Polypharmacy, low level of physical activity, 3MS score, and abnormal IADL score were significantly different between groups (*P* ≤ 0.003; Table 1). A lower prevalence of polypharmacy, low level of physical activity, and abnormal IADL score were reported in individuals without late-life depressive symptomatology and MCR compared to those with late-life depressive symptomatology only (*P* ≤ 0.01). Individuals with MCR had a lower 3MS score and a higher prevalence of abnormal IADL score compared to those without late-life depressive symptomatology and MCR (*P* ≤ 0.01). The lowest 3MS score and the highest prevalence of abnormal IADL score were reported in individuals with late-life depressive symptomatology and MCR, the difference was significant compared to those without late-life depressive symptomatology and MCR (*P* ≤ 0.01). Individuals with late-life depressive symptomatology and MCR had a higher prevalence of abnormal IADL score compared to those with late-life depressive symptomatology (*P* ≤ 0.01).

**Table 1:** Baseline characteristics of participants grouped according to their late-life depressive symptomatology and MCR status (*n* = 1,098).

	**Participants**				***P*-value^‡^**
	No late-life depressive symptomatology^*^^†^ and no MCR **(*n* = 771)**	Late-life depressive symptomatology^*^^†^ ** (*n* = 341)**	**Motoric cognitive risk Syndrome^†^** ** (*n* = 20)**	**Late-life depressive symptomatology plus MCR** ** (*n* = 26)**	
Age (years), mean ± SD	73.6 ± 4.1	74.1 ± 4.1	74.9 ± 4.1	75.1 ± 4.0	0.058
Female, n (%)	354 (49.8)	197 (57.8)	9 (45.0)	13 (50.0)	0.095
Overweight/obesity^¶^, *n* (%)	508 (71.4)	245 (71.8)	14 (70.0)	19 (73.1)	0.995
Polypharmacy^§^, *n* (%)	282 (39.7)	183 (53.7)^a^	12 (60.0)	16 (61.5)	**≤0.001**
Past history of falls, *n* (%)	117 (16.5)	72 (21.1)	6 (30.0)	4 (15.5)	0.143
Low level of physical activity^#^, n (%)	210 (29.5)	138 (40.5)^a^	8 (40.0)	11 (42.3)	**0.003**
3MS score (/100), mean ± SD	94.6 ± 4.0	94.4 ± 4.2	92.3 ± 4.5^b^^,^^d^	89.9 ± 4.5^c^^,^^e^	**≤0.001**
IADL score <4 (/4), *n* (%)	73 (10.3)	55 (16.1)^a^	5 (25.0)^b^	13 (50.0)^c^^,^^e^	**≤0.001**
Incident dementia^**^, *n* (%)	12 (1.7)	10 (2.9)	2 (10.0)^b^	5 (19.2)^c^^,^^e^	**≤0.001**

*3MS, modified mini-mental state; SD, standard deviation; IADL, instrumental activity daily living*.

* *30-item Geriatric Depression Scale score >10/30*.

† *Exclusive (i.e., only participants with the condition)*.

‡ *Multiple comparisons based on ANOVA Kruskal–Wallis or Chi-square test, as appropriate*.

¶ *Body mass index ≥ 25 kg/m^2^*.

§ *Number of therapeutic drugs daily taken ≥ 5*.

# *Score of the Physical Activity Scale for Elderly below the lowest tertile (i.e., < 69.1 for female and < 87.7 for male)*.

** *Overall incident of dementia diagnosed with Modified Mental State score ≤ 79/100 and instrumental activity daily living score <4/4 at each annual visit after the baseline assessment*.

a *Comparison of participants without late-life depression and motoric cognitive risk syndrome (MCR) with participants with significant late-life depression (i.e., <0.01)*.

b *Comparison of participants without late-life depression and MCR of participants with a significant MCR (i.e., <0.01)*.

c *Comparison of participants without late-life depression and MCR of participants with combined late-life depression and significant MCR (i.e., <0.01)*.

d *Comparison of participants with late-life depression and participants with significant MCR (i.e., <0.01)*.

e *Comparison of participants with late-life depression and participants with combined late-life depression and significant MCR (i.e., <0.01)*.

*Significant values of P (i.e., <0.01) are indicated in bold*.

**Figure 1:**
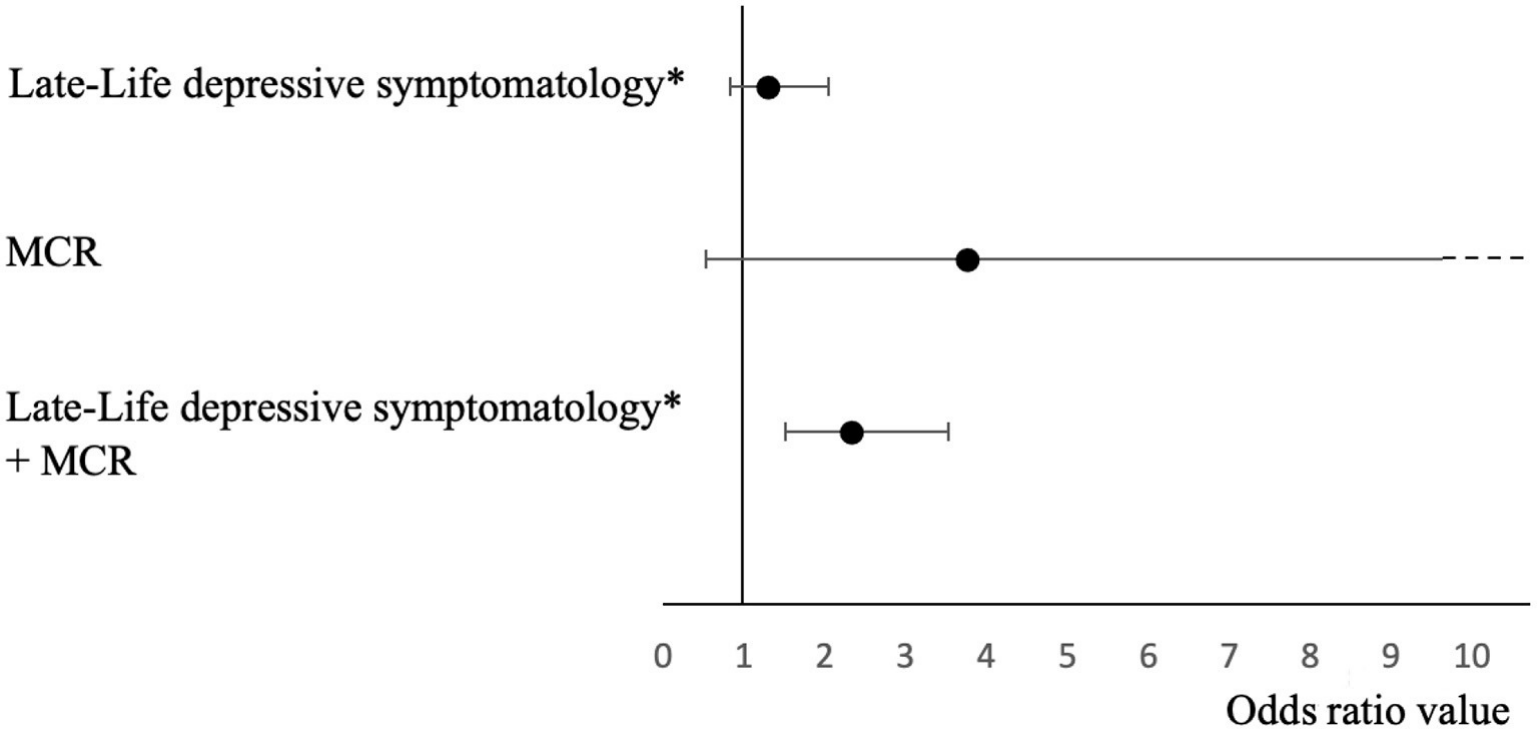
Logistic regressions showing odds ratios and confidence intervals of the association of late-life depressive symptomatology, MCR, and their combination with overall incident dementia adjusted for participant's baseline characteristics (*n* = 1,098). *score >5/30; MCR, motoric cognitive risk syndrome.

The overall incidence of dementia was different between groups (*P* ≤ 0.001), the highest incidence was shown in individuals exhibiting both late-life depressive symptomatology and MCR. Individuals with MCR only and with late-life depressive symptomatology and MCR had a higher incidence compared to those without late-life depressive symptomatology and MCR (*P* ≤ 0.01). Individuals with late-life depressive symptomatology and MCR had a higher incidence of dementia compared to those with late-life depressive symptomatology (*P* ≤ 0.01). The combination of late-life depressive symptomatology and MCR at baseline was associated with significant overall incident dementia (odds ratio (OR) = 2.31 with 95% confidence interval (CI) = [1.51–3.52] with *P* ≤ 0.001 and R-Square = 0.224) but not for MCR only (*OR* = 3.75 with 95% CI = [0.53–26.56] with *P* = 0.186 and R-Square = 0.285) or late-life depressive symptomatology (OR = 1.29 with 95% CI = [0.82–2.04] with *P* = 0.276 and R-Square = 0.132) only (Figure 1).

## Discussion

The results showed that the combination of late-life depressive symptomatology and MCR is associated with incident dementia but not late-life depressive symptomatology and MCR only in the NuAge participants.

The risk for incident dementia increased in participants combining late-life depressive symptomatology and MCR in the present study. This increased risk was higher compared to participants who had only one symptomatology (around 2.3-fold), and similar compared to the risk reported in individuals with MCR in previous original studies and a recent meta-analysis (pooled estimated ratio for risk of incident dementia in participants with MCR at baseline compared to those without the MCR = 2.5 with 95% CI = [1.75–2.39]) (Sekhon et al., 2019b). This result highlights that, first, an overlap between late-life depressive symptomatology and MCR is possible and, second, that this overlap does not interfere with the risk for dementia, suggesting a complex interplay between depressive symptomatology and MCR. This effect may be of clinical utility for the prevention of dementia. For instance, over half a million Canadians are living with dementia (Beauchet et al., 2021). The health care system in Canada, like others, is ill-equipped to deal with the resulting staggering costs of dementia. One of the three key objectives of the primary prevention strategy of Canada is to reduce the rate of conversion to dementia. Better understanding the association between late-life depressive symptomatology and MCR is in line with this objective. Indeed, this could improve the detection of individuals at risk for dementia at a population level and, consequently, orient appropriate interventions.

We did not report an association between MCR only and overall incident dementia, whereas we showed that this incidence of dementia was higher in participants with MCR only compared to those without MCR and late-life depressive symptomatology. These mixed results have been reported in a recent meta-analysis (Sekhon et al., 2019b). One study selected in this meta-analysis showed no significant association with incident dementia, similar to our study (Kumai et al., 2016). An explanation for the absence of a significant association between MCR only and overall incident dementia in our study may be the good health condition at the baseline assessment of the NuAge participants. Another explanation may be the classification of individuals. Being in the group of participants with MCR only means that participants with MCR and late-life depression were excluded from this group. Thus, the group of MCR patients has been split into two subgroups (i.e., MCR only and MCR with late-life depressive symptomatology) that may explain the absence of significant association between MCR only and incident dementia. Similar to MCR only, late-life depressive symptomatology only was not associated with an increased risk for dementia. Two reasons may explain this result. First, the 30-item GDS score is a way to identify an individual with depressive symptomatology. The final diagnosis of depression must be performed by a clinician. Second, similar to the inconclusive association between MCR only and the incident dementia, the good health condition of the NuAge participants may influence the association of late-life depressive symptomatology and dementia.

Some limitations of the present study need to be underscored. First, the NuAge population was composed of relatively healthy older adults that could prevent the generalization of the results of the present study. Second, the prevalence of MCR (1.8%) and the combination with depressive symptomatology (2.4%) was low, which may lead to a lack of power to show an association with incident dementia in one hand and bringing some degree of uncertainty on their association with incident dementia on the other hand. More studies with a greater variety of health status in older adults are, therefore, required to further confirm this association. Third, although we were able to control for many characteristics likely to modify the association, residual confounding might still be present. As confounding factors can impact both the magnitude and the direction of the association, it is difficult to speculate on the impact of residual confounding factors on the associations found in the study. Fourth, the diagnosis of dementia may be underestimated that may explain its low incidence. Indeed, this diagnosis is usually based on an interdisciplinary meeting and more exhaustive information. In our case, we used only the threshold for dementia of 3MS combined with abnormal simplified IADLs score.

## Conclusions

Our study showed that the combination of late-life depressive symptomatology and MCR, but not its components, is associated with incident dementia in Quebec community-dwelling older adults. The results suggest an interplay between late-life depressive symptomatology and MCR increasing the risk for dementia.

## Data Availability Statement

The data analyzed in this study is subject to the following licenses/restrictions: Access to NuAge Database can be obtain by contacting the NuAge team via NuAge-cdrv@usherbrooke.ca. Requests to access these datasets should be directed to the NuAge team, NuAge-cdrv@usherbrooke.ca.

## Ethics Statement

The studies involving human participants were reviewed and approved by The Research Ethics Boards (REB) of the University Institutes of Geriatrics of Sherbrooke and Montreal (Quebec, Canada). The patients/participants provided their written informed consent to participate in this study.

## Author Contributions

OB and GA: conceived and designed the experiments. PG and JM: cohort data collection. OB, HS, CL, and GA: analyzed and interpreted the data. OB: contributed reagents, materials, analysis tools, and data. OB, HS, and CL: writing of the manuscript. PG, JM, and GA: revision of the manuscript. All authors contributed to the article and approved the submitted version.

## Funding

The NuAge Study has been funded by the Canadian Institutes of Health Research (CIHR; MOP-62842). The NuAge Database and Biobank are supported by the Fonds de recherche du Québec (FRQ; 2020-VICO-279753), the Quebec Network for Research on Aging funded by the FRQ-Santé, and by the Merck-Frosst Chair funded by La Fondation de l'Université de Sherbrooke.

## Conflict of Interest

The authors declare that the research was conducted in the absence of any commercial or financial relationships that could be construed as a potential conflict of interest.

## Publisher's Note

All claims expressed in this article are solely those of the authors and do not necessarily represent those of their affiliated organizations, or those of the publisher, the editors and the reviewers. Any product that may be evaluated in this article, or claim that may be made by its manufacturer, is not guaranteed or endorsed by the publisher.
